# Associations between neighborhood violence during pregnancy and birth outcomes: evidence from São Paulo’s Western Region Birth Cohort

**DOI:** 10.1186/s12889-021-10900-y

**Published:** 2021-05-05

**Authors:** Angélica Carreira dos Santos, Alexandra Brentani, Günther Fink

**Affiliations:** 1 Department of Pediatrics, University of São Paulo Medical School, Av. Dr. Enéas Carvalho de Aguiar, 647, São Paulo, CEP-01246-904 Brazil; 2 Department of Epidemiology and Public Health University of Basel, Swiss Tropical and Public Health Institute, Basel, Switzerland

**Keywords:** Violence, Pregnancy, Child health, Birth weight, Social determinants of health

## Abstract

**Background:**

Low birth weight and prematurity remain leading causes of infant mortality and morbidity globally. Although extensive literature has highlighted the importance of socioenvironmental characteristics for birth outcomes, the role of indirect violence on health remains fairly understudied.

**Methods:**

Using geocoded birth records from the ongoing Western Region Birth Cohort (*Região Oeste Coorte* – ROC-Cohort) of infants born between 2012 and 2014 and geocoded crime reports, we assessed the associations between exposure to violent crimes during pregnancy within a 1-km radius of the mother’s residence and low birth weight, preterm delivery, and being born small-for-gestational-age. Violent crime exposure was categorized into quintiles. Multivariate logistic regressions were used to examine the associations between violence exposure and birth outcomes. Models were adjusted for sex, maternal age and education, socioeconomic status, and risk factors such as hypertension, diabetes, smoking, and drinking during pregnancy.

**Results:**

Among the 5268 children included, the average crime exposure during the first two trimesters of pregnancy ranged from 0.44 violent crimes in the least exposed quintile to 12.74 crimes in the most exposed. Compared to children with the lowest violence exposure, children in the highest exposure quintile had higher odds of being born small-for-gestational-age (1.41[1.06–1.89]), preterm (1.35[1.01–1.80]), and low birth weight (1.42[1.03–1.98]). While socioeconomic status and maternal education were positively associated with lower violence exposure, no associations were found between these characteristics and birth outcomes.

**Conclusions:**

Higher exposure to violent crimes in the close vicinity of pregnant women’s residence is associated with substantial increases in the odds of adverse birth outcomes. Policies to improve neighborhood safety can potentially contribute not only to the short-term wellbeing of populations but may also have large social, economic, and health benefits in the long term.

**Supplementary Information:**

The online version contains supplementary material available at 10.1186/s12889-021-10900-y.

## Background

Low birth weight accounts for more than 80% of all neonatal mortality globally [1]. In 2015, more than 1 million children under 5 died because of preterm birth complications globally [2]. Neonates born with low birth weight – about two-thirds of which are preterm [1] - face higher morbidity risks, increased risk of stunting, increased risk of long-term development impairment, as well as increased vulnerability to chronic disease [1–7].

Violence is a growing public health concern with immediate and long-term influences on population health [8–10]. Contrary to some positive trends observed in high-income countries (HIC) in recent years [10], homicide rates have been increasing in Latin America over the past years [11]. An estimated homicide rate of 28.5 implies that Latin America’s crime rates are approximately 8 times higher than those seen in HICs on average and 14 times those seen in low-middle-income countries (LMICs) in the Western Pacific Region [11].

Exposure to violence has been consistently associated with mental health problems and high-risk behaviors such as alcohol, smoking, and drug abuse, which threaten the wellbeing of both adult and child populations [9, 12], and which are also well-known risk factors for low birth weight, small-for-gestational-age, and preterm birth. Fear of crime can trigger anxiety and feelings of isolation, victimization, powerlessness, and normlessness [13], particularly among women [14]. Moreover, stress may indirectly increase the risk of adverse birth outcomes through the development of depressive symptoms and low commitment to prenatal care [15–18], and inadequate dietary intake [19].

Chronic exposure to such environmental stress can trigger neural or neuroendocrine responses, change the functioning of the hypothalamic-pituitary-adrenal (HPA) axis and lead to increased allostatic load [20]. Stress may also increase the placental secretion of corticotrophin-releasing hormones, which increase prostanoids and stimulate uterine contractions and can induce preterm delivery [17, 20–23]. Studies have also shown that increased activation of the maternal HPA axis and sympathetic nervous systems can induce restrictions in uterine vasculature, resulting in intrauterine growth restriction or preterm birth [7].

A growing body of literature has tried to link external violence’s to adverse birth outcomes at the area level [24–33]. The large majority of studies are based on neighborhood comparisons in the United States and likely allow only limited insight into the impact of violence in low-income settings due to the large structural differences in neighborhoods and public support systems. The few studies that have been conducted in low-income and high crime exposure areas have mostly focused on sociopolitical violence and generally found sizeable increases in adverse birth outcomes in response to major violence outbreaks [24, 34, 35]. This is consistent with other studies focusing on more extreme forms of violence, such as terrorist attacks and civil/war conflicts, generally finding large increases in the risk of pregnancy complications outcomes after such major events [24, 36, 37]. Moreover, the effects of violence on adverse birth outcomes might differ from areas where violence is rare and areas where violence is considered endemic, having each additional violent crime smaller adverse consequences on birth outcomes due to the process of adaptation [27].

In this paper, we aim to explore the relationship between exposure to violent crime in close vicinity and three of the most common adverse birth outcomes: preterm birth, small-for-gestational-age, and low birth weight in one of the largest metropolitan areas of the world [38]. Using a novel data set, we aim to improve the understanding of the health consequences of violent crimes on pregnant women and their offspring in a low-income population. Our initial hypothesis is that the occurrence of violent crime close to home is associated with these adverse birth outcomes, independent of maternal and socioenvironmental characteristics.

## Methods

### Study design

This study was designed as a retrospective cross-sectional study linking data collected as part of a prospective cohort study to geocoded administrative data on violent crime.

### Study setting

This study was conducted in the municipality of São Paulo, Brazil. In São Paulo State, the proportion of preterm births increased from 7.7% in 2004 (7.7%) to 12.4% in 2013 [39]. At the same time, preterm birth increased from 8.6% in 2004 to 11.6% in 2013 in the São Paulo municipality. In 2016, preterm rates were 10.9 and 10.7% in São Paulo State and São Paulo municipality, respectively [39]. Regarding low birth weight, both State and municipality had low variation across time, considering the available data, with the highest proportion observed in 2012, 9.5 and 9.7%, respectively [40]. Data on children born small for gestational age is not publicly available for either area.

Like other LMICs, Brazil has experienced a rapid and mostly unmanaged urbanization process in the past decades, accompanied by large increases in social and economic inequality [41, 42]. The rates of homicides in Brazil rose steadily over the past years, reaching a record level of 31.6 homicides per 100,000 inhabitants in 2017 [43]. With 10.3 homicides per 100,000 inhabitants, the São Paulo municipality had one of the lowest homicide rates in Brazil in 2017 overall, but still experienced very high crime rates in areas of low socioeconomic development [43]. In the Western Region of São Paulo, where this study was conducted, homicide rates ranged between 5 and 20 homicides per 100,000 inhabitants across neighborhoods [44].

### Study population

The São Paulo Western Region Birth Cohort (*Região Oeste Coorte* – ROC-Cohort) enrolled locally resident babies born at the University Hospital of the School of Medicine of the University of São Paulo between April 1, 2012, and March 31, 2014. The study’s main objective was to evaluate the impact of environmental and social determinants of health on child development. A total of 7066 births occurred in the period, and 6207 mother-child pairs who resided in the studied setting were enrolled in the cohort. Children were followed-up at 6 months, 12 months, and 36 months. The cohort is still active, and the 72 months (6 years) follow-up was paused due the ongoing COVID-19 pandemic. The follow-up rounds targeted all enrolled children who were alive at the time of the follow up. Assessments involved child development, mental health, health habits and status, parenting practices and general living conditions. A full description of the cohort can be found elsewhere [45].

### Data

Hospital electronic birth records were available for all children in the cohort. The electronic medical registry includes birth characteristics such as type of delivery, gestational length, weight at birth, and others. During the postpartum hospital stay, all mothers were invited to complete a short-structured questionnaire administered by a trained interviewer to collect information on socio-demographic characteristics and health during pregnancy. The questionnaire was applied after maternal consent or a parent if the mother was too young to consent. The questionnaire can be found in Supplementary file 1, and the rates of interview and process can be found in the cohort profile [45].

### Outcomes

The analysis focused on three adverse birth outcomes: low birth weight (LBW), preterm delivery (PT), and small-for-gestational-age (SGA). Birth weight was measured by the Hospital’s neonatology team immediately after birth using standard hospital equipment. Gestational length in weeks was estimated using the New Ballard Score [46]. LBW was defined as birth weight < 2500 g, and SGA was defined as weight-for-gestational age < 10th percentile based on the Intergrowth-21st growth reference Tables [47].

### Exposure – violence in the neighborhood

Data on violence is routinely collected and made publicly available by the Secretariat of Public Safety of the State of São Paulo and can be accessed on www.ssp.sp.gov.br. The system collects detailed information on all willful murders, femicides (gender-based murder), robberies followed by death, bodily injuries followed by death, deaths resulting from police intervention, suspicious deaths, fatal vehicle accidents, and cellphone and car theft occurring in São Paulo State. Data on other crimes such as intimate partner abuse (physical, emotional, financial), rape, and child sexual assault are not collected in this system.

Each reported incident record contains the date, time, and address of the crime. Following most of the external violence literature, we focused on violent crime in our analysis, which includes willful murder, robbery followed by death, bodily injury followed by death and death resulting from police intervention but excludes other crimes such as robberies without injuries. Feminicide was not included in the analysis because this data was only available after 2015.

We extracted data on violent crimes between 2011 and 2014 to cover all ROC-Cohort children’s pregnancy period. The address of each reported crime was geocoded with latitude and longitude coordinates using the *ggmap* package [48], and a point-layer for each time point (month-year) was generated using R map tools [49]. The maternal residential address was collected at birth and geocoded as an additional point-layer. Each residential address was treated as a centroid point. We then computed the number of crimes within a 1-km spatial buffer by day, month, and year. Exposure to violence during pregnancy was estimated as the sum of violent crimes in the first two trimesters of pregnancy. Violence in the third trimester was not considered because total exposure to violence after week 24 of gestation directly depends on gestational length in weeks (the outcome variable). The sum of violent crimes within 1-km of each mother’s residence during the first two trimesters was categorized into five equally sized quintiles for analysis. This categorization was chosen to allow us to compare the 20% of births with the lowest external violence exposure to families in higher exposure quintiles. We provide a detailed discussion of the exposure differences between these quintiles below.

### Statistical analyses

We first computed the average number of crimes for each of the exposure quintiles. Next, we tested crude associations of exposure to violence quintiles with each outcome (LBW, SGA, and PT birth). In the fully adjusted multivariable models, we included all covariates highlighted in the existent literature as risk factors for adverse birth outcomes, including maternal age (< 20, > = 35), educational level (incomplete primary, complete secondary and tertiary), alcohol consumption (yes/no), smoke (yes/no), diabetes (yes/no), hypertension (yes/no) and experience of physical violence (yes/no) during pregnancy – reported by the mother in the postpartum questionnaire, and socioeconomic (SES) index. The construction of a socioeconomic (SES) index was based on the approach proposed by Vyas and Kumaranayake [50], which relies on the use of principal component analysis (PCA) of the selected variables. The SES index was based on questions regarding asset holdings such as cars, family income, and household characteristics. The PCA’s first principal component was then divided into five SES quintiles - low, medium-low, medium, medium-high, and high SES status. Maternal age was categorized into adolescence (< 20), core reproductive age (22–34) and ages > 35.

Given that the postpartum questionnaire was not available for the whole sample, we initially screened the dataset for missing data and patterns of non-responsiveness in the postpartum questionnaire using t-test and chi-square to test if the assumptions of missing at random (MAR) were met. The reasons for not completing the postpartum questionnaire vary from being too young to consent to being discharged before the interview could be completed. The details can be found elsewhere [45]. As the assumptions of MAR in our sample were met, we implemented the MICE to complete the missing data in the postpartum questionnaires to all cohort participants. We implemented multiple imputations by chained equations (MICE) with a fully conditional specification of prediction equations using variables that potentially predicted nonresponse or the outcome. The MICE method accounts for statistical uncertainty in the imputations and ensures more reliable inference in settings with missing data. All imputed models had a relative efficiency higher than 99%.

We performed the MICE using Stata’s *mi* package [51]. The variables used in the MICE models included exposures (e.g., violence during pregnancy), birth outcomes (PT, LBW and SGA), maternal characteristics (e.g., age, socioeconomic, education), pregnancy variables (e.g., diabetes, hypertension), and neonatal variables (e.g., birth weight, length, and sex). We generated 50 independent datasets (M = 50) and followed Rubin’s rules to aggregate results across imputed datasets [52]. All analyses were performed using Stata version 14 [53]. Further details on these imputations are provided in Table S1 (Supplementary file 2).

## Results

Among the 6207 children in the ROC-Cohort, 44 were stillbirths and excluded from the analysis. The likelihood of being stillborn was not associated with violence exposure during pregnancy in our sample (0.99; 95%CI: 0.92–1.06). Additionally, 895 mother-child dyads were excluded due to an address outside of the studied setting. Overall, 5268 children and their mothers met the inclusion criteria for this study.

Mothers who did not respond to the postpartum questionnaire (*n* = 2008) did not differ from those who completed (*n* = 3260) the survey with respect to the type of birth, gestational length category, birth weight and length, and child sex.

398 (7.8%) births were classified as SGA, 369 (7.0%) as PT, and 355 (6.7%) as LBW. Table 1 shows the frequency of each type of crime during pregnancy by exposure quintile. From 2011 to 2014, there were 8504 violent crimes in the São Paulo municipality – only 13 of these could not be geolocated (0.15%). 339 (4%) of all crimes in the municipality were located in the Butanta-Jaguaré area (where all children of the cohort reside). During the first two trimesters, the mean number of crimes occurring within a 1-km radius from home was 4.2 (SD ± 4.8). The large majority of violent crimes were murders; death resulting from police intervention was the second most common type of violent crime. Women with the lowest exposure quintile were on average exposed to 0.45 (SD ± 0.5) violent crimes, while women in the fifth quintile were exposed on average to 12.7 (SD ± 4.5) violent crimes. Only 19% of women were not exposed to any violent event in a 1 km radius during pregnancy; some women in the top exposure quintile experienced more than 30 violent crimes during the first two trimesters.

**Table 1:** Mean number of violent crime type in pregnancy per exposure quintile.

	1st quintile	2nd quintile	3rd quintile	4th quintile	5th quintile
	Mean ± SD	Mean ± SD	Mean ± SD	Mean ± SD	Mean ± SD
Murder	0.39 ± 0.49	1.71 ± 0.67	2.64 ± 0.71	4.34 ± 1.38	9.08 ± 2.85
Assault followed by death	0.26 ± 0.65	0.26 ± 0.65	0.29 ± 0.65	0.48 ± 0.90	0.65 ± 1.50
Bodily injury followed by death	0.01 ± 0.08	0.02 ± 0.12	0.01 ± 0.10	0	0
Death resulting from police intervention	0	0.01 ± 0.16	0.06 ± 0.34	0.22 ± 0.64	3.02 ± 2.79

SD: standard deviation

**Fig. 1:**
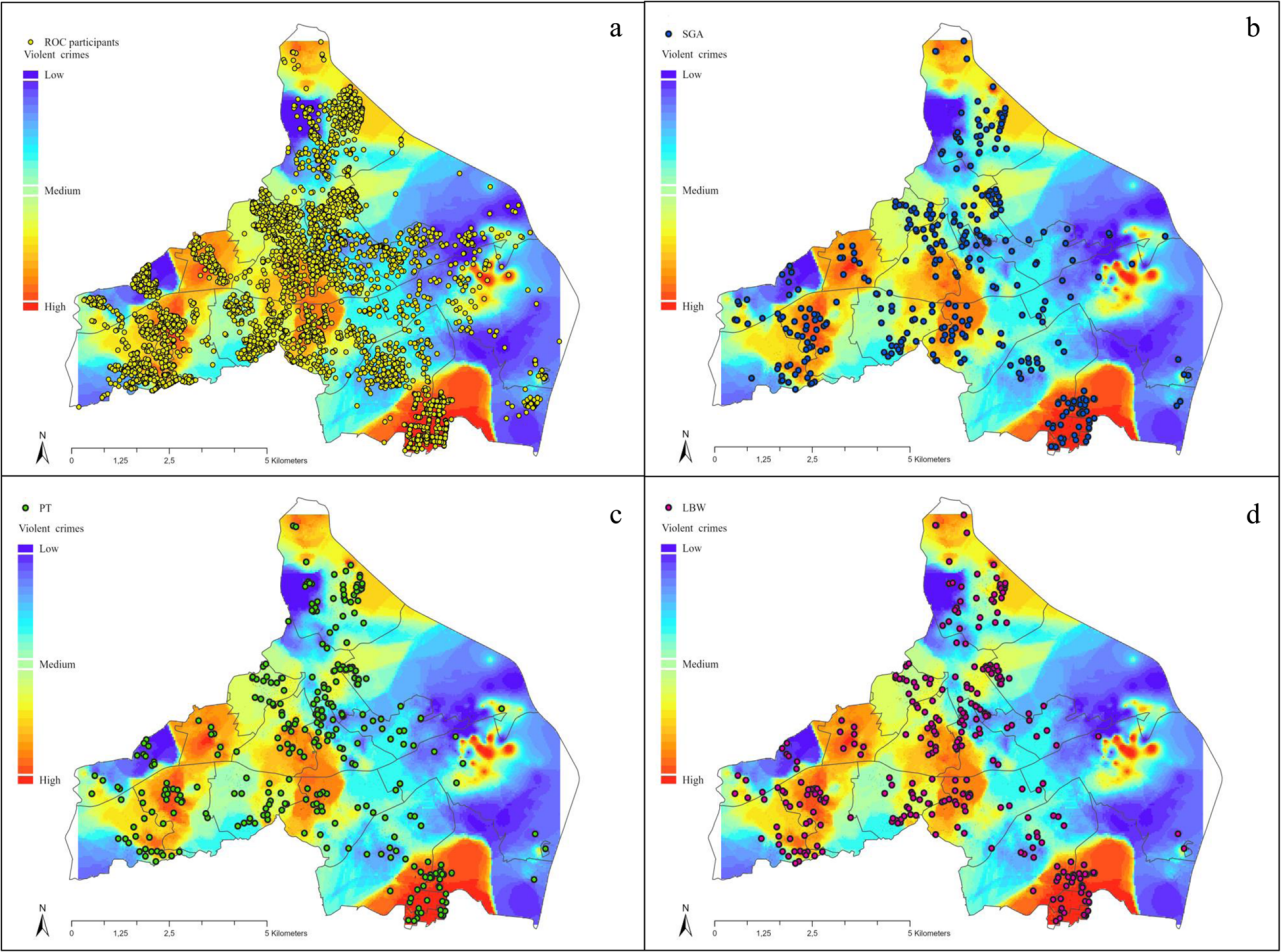
Heatmap of the Butantã-Jaguaré Region of São Paulo showing the distribution of violent crimes during pregnancy. Blue indicates lower number of violent crimes incidents and red higher. The point layers show the geographic distribution of ROC residences (**a**), infants born small for gestational age (**b**), preterm births (**c**), and low birth weight (**d**)

Figure 1 shows the geographic distribution of the outcomes and violent crimes in the studied area. Areas colored in red represent the highest crime exposure; blue areas are safest. Panel A of Figure one shows the overall spatial distribution of children in the study area. Panels B, C, and D illustrate the spatial distribution of the three adverse birth outcomes.

The prevalence of SGA, PT and LBW were 6.7, 7.9, and 9.5% (SGA), 6.5, 6.4, and 6.3% (PT), and 5.4, 7 and 7.5% (LBW) in the first, third, and fifth exposure quintiles, respectively. Table 2 shows the distribution of SGA, PT, and LBW birth outcomes and family characteristics by exposure quintile using the raw (non-imputed) data. The number of available data points for each variable as well as additional information on the MICE can be found in Supplementary file 2. Table 2 also shows the bivariate analysis between continuous violent crimes quintiles (range 1 to 5), and birth outcomes, and child and family characteristics. In bivariate analysis LBW was strongly associated with crime exposure (1.09; 95%CI: 1.02–1.17, *p* = 0.014), while PT and SGA displayed positive but not statistically significant associations with violent crimes. Upper middle (0.91; 95%CI: 0.85–0.98, *p* = 0.013) and high (0.83; 95%CI: 0.77–0.89, *p* <  0.001) SES status was negatively associated with violent crimes. Physical violence during pregnancy reported in the postpartum questionnaire was also negatively associated with violent crimes (0.79; 95%CI: 0.66–0.95, p = 0.013).

**Table 2:** Birth outcomes and child and family characteristics before multiple imputation by external violence exposure quintile, and the association between the pooled violent crimes quintiles and birth outcomes and child and family characteristics.

Violent crimes quintile Number of occurrences	1st quintile Low: 0–1		2nd quintile Lower-meddle: 2		3rd quintile Middle: 3		4th quintile Higher-middle: 4–7		5th quintile High: 8–37		OR^*^ (95%CI)	p
Variables	n	%	n	%	n	%	n	%	n	%		
SGA (*N* = 5080)												
Yes	116	6.7	78	9	36	7.9	81	7.4	87	9.5	1.06 (1.00–1.14)	0.062
PT (*N* = 5268)												
Yes	116	6.5	68	7.5	30	6.4	94	8.3	61	6.3	1.02 (0.95–1.09)	0.594
LBW (*N* = 5268)												
Yes	96	5.4	66	7.3	33	7.0	88	7.7	72	7.5	1.09 (1.02–1.17)	0.014
Fetal sex (*N* = 5268)												
Female	898	50.2	449	49.5	246	52.1	589	51.8	469	48.7	1.00 (0.96–1.03)	0.937
Maternal age (*N* = 5268)												
< 20 years	381	21.3	236	26.0	93	19.7	274	24.1	239	24.8	1.03 (0.99–1.07)	0.157
20–35 years	1191	66.6	561	61.9	327	69.5	745	65.5	626	64.9		Ref.
> 35 years	216	12.1	110	12.1	52	11.0	118	10.4	99	10.3	0.96 (0.90–1.01)	0.125
Skin color (*N* = 5267)												
White	1080	60.4	575	63.4	286	60.7	690	60.7	593	61.5		Ref.
Non-white	707	39.6	332	36.6	186	39.4	447	39.3	371	38.5	1.00 (0.96–1.03)	0.888
SES status (*N* = 2709)												
Low	144	16.0	92	19.0	45	18.0	135	23.7	128	24.6	1.08 (1.00–1.16)	0.045
Lower-middle	225	25.1	126	26.0	66	27.9	150	26.4	156	30.0		Ref.
Middle	126	14.0	84	17.3	40	16.9	91	16.0	79	15.2	0.98 (0.91–1.06)	0.605
Upper-middle	213	23.7	105	21.7	48	20.3	79	15.2	104	20.0	0.91 (0.85–0.98)	0.013
High	190	21.2	78	16.1	38	16.0	104	20.0	53	10.2	0.83 (0.80–0.89)	< 0.001
Education (*N* = 3256)												
Incomplete primary	154	13.2	90	16.2	44	15.8	101	14.9	109	18.9	1.07 (1.01–1.14)	0.026
Complete secondary	934	80.0	439	79.1	216	77.7	551	81.0	452	78.5		Ref.
Tertiary	79	6.8	26	4.7	18	6.5	28	4.1	15	2.6	0.83 (0.13–0.19)	< 0.001
Diabetes (*N* = 3246)												
Yes	36	3.1	16	2.9	6	2.2	17	2.5	16	2.8	0.95 (0.83–1.09)	0.500
Hypertension (*N* = 3249)												
Yes	130	11.2	40	7.2	21	7.6	65	9.6	51	8.9	0.95 (0.88–1.03)	0.189
Smoke in pregnancy (*N* = 3240)												
Yes	161	14.0	86	15.2	37	13.3	90	13.22	83	14.4	0.99 (0.93–1.06)	0.802
Drink in pregnancy (*N* = 3245)												
Yes	161	13.9	75	13.5	40	14.4	79	11.6	66	11.4	0.94 (0.88–1.01)	0.079
Depression (*N* = 3251)												
Yes	34	2.9	13	2.3	3	1.1	23	3.4	9	1.6	0.93 (0.81–1.07)	0.329
Physical violence (*N* = 3236)												
Yes	27	2.3	14	2.5	4	1.5	6	0.9	7	1.2	0.79 (0.66–0.95)	0.013

Quintiles categorized violent crimes within a 1-km from participants’ home address; OR: odds ratio; 95% CI: 95% confidence interval; SGA: small for gestational age; PT preterm; LBW: low birth weight; SES: socioeconomic status. * For the variables: Maternal age, SES status and Education, the values represent relative risk-ratios (RRR)

**Fig. 2:**
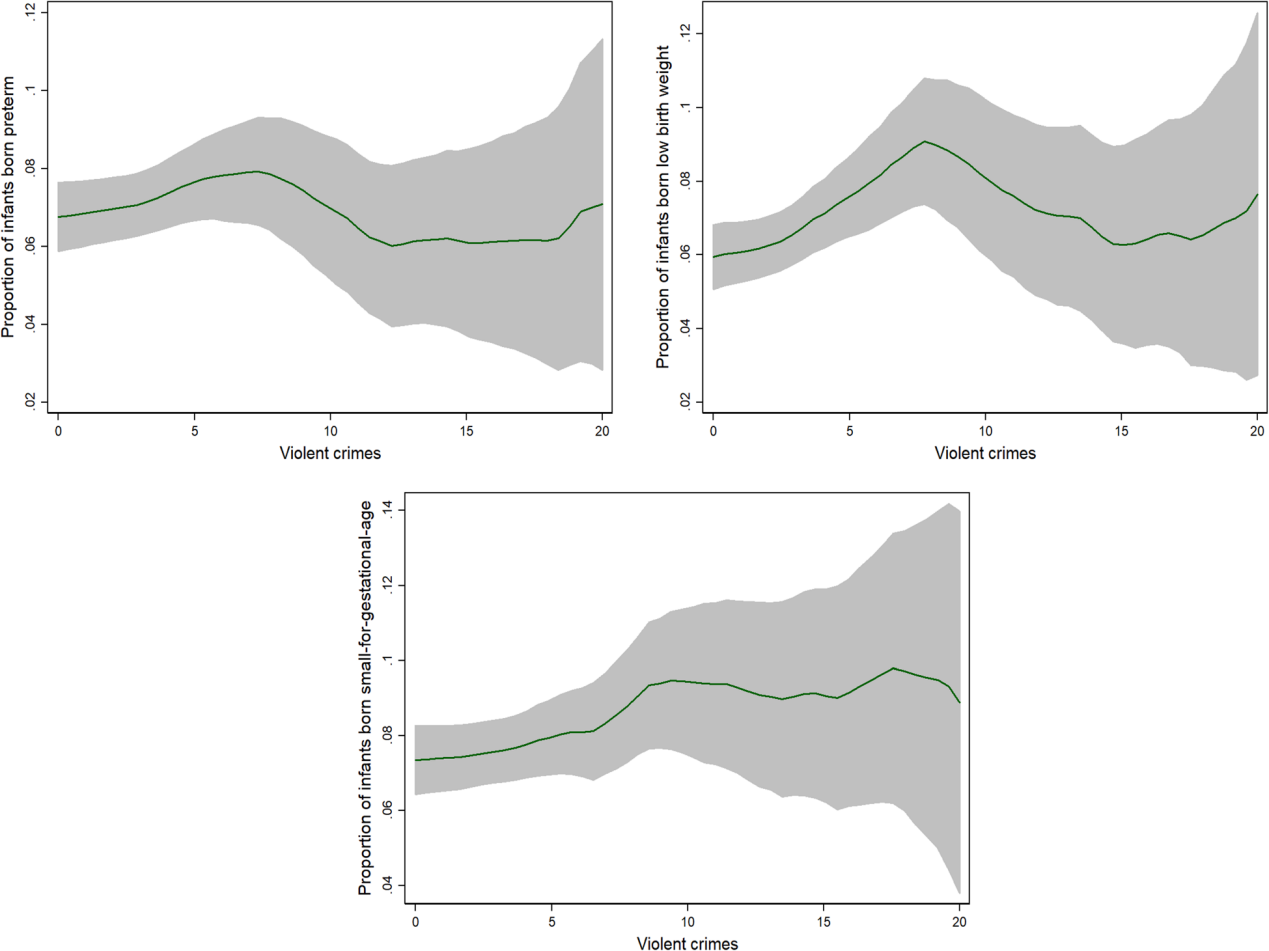
Exposure to violent crime and average birth outcomes. The figure shows log polynomial smoothed relationship between violent crime exposure and preterm birth (**a**), low birth weight (**b**), and small-for-gestational-age (**c**)

Figure 2 shows the bivariate relationship between exposure to violent crime and the proportion of infants born SGA (panel a), preterm (panel b) and LBW (panel c). Table 3 shows the unadjusted and adjusted associations between violent crime exposure during pregnancy and adverse birth outcomes. All models were adjusted using maternal age, child sex, education, and socioeconomic status. Relative to children from the lowest violence quintile, living in the highest violence quintile was associated with a 46% increase in SGA odds in the unadjusted model (1.46; 95%CI: 1.10–1.93). After adjustment for covariates, risk factors, and SES status, the estimated odds ratio (OR) slightly decreased (1.41; 95%CI: 1.06–1.89). In the adjusted model, PT birth odds increased nonlinearly (Fig. 2) with violence exposure, varying the estimated ORs from 1.16 (95%CI: 0.84–1.59) on quintile 2, 1.35 (95%CI: 1.01–1.80) on quintile 4, and 1.01 (95%CI: 0.72–1.40) on quintile 5. The estimated risk of LBW increased almost linearly with exposure quintiles, with an estimated OR of 1.43 (95%CI: 1.03–1.98) for the top quintile. In terms of the included covariates, smoking during pregnancy was the most consistent risk factor for all birth outcomes analyzed in this study, with estimated ORs of 1.83 for SGA (95%CI: 1.32–2.55), 2.04 for PT birth (95%CI: 1.47–2.82), and 2.89 for LBW (95%CI: 2.07–4.03). Physical violence reported by the mother during pregnancy (in the post-partum interview) was associated with more than twice the odds of LBW (2.12; 95%CI: 1.03–4.36). SES and maternal education were not associated with the outcomes in our analysis.

**Table 3:** Unadjusted and adjusted analyses between violent crimes exposure during pregnancy by quintiles, and birth outcomes.

	Unadjusted			Adjusted^*^		
	OR	95%IC	p	OR	95%IC	p
**Small-for-gestational-age**						
Violent crimes						
1st quintile	Ref.					
2nd quintile	1.36	1.02–1.83	0.039	1.34	1.00–1.80	0.054
3rd quintile	1.20	0.82–1.75	0.349	1.19	0.81–1.75	0.373
4th quintile	1.12	0.84–1.50	0.418	1.11	0.84–1.50	0.449
5th quintile	1.46	1.10–1.93	0.009	1.41	1.06–1.89	0.019
Physical violence				1.07	0.43–2.65	0.896
Depression				0.42	0.13–1.36	0.147
Diabetes				0.70	0.27–1.82	0.468
Hypertension				1.27	0.82–1.95	0.286
Smoke				1.83	1.32–2.55	< 0.001
Drink				1.43	0.99–2.05	0.055
**Preterm birth**						
Violent crimes						
1st quintile	Ref.					
2nd quintile	1.17	0.86–1.59	0.327	1.16	0.84–1.59	0.367
3rd quintile	0.98	0.65–1.48	0.918	1.01	0.66–1.54	0.958
4th quintile	1.29	0.98–1.72	0.070	1.35	1.01–1.80	0.040
5th quintile	0.97	0.71–1.34	0.870	1.01	0.72–1.40	0.969
Physical violence				1.62	0.77–3.44	0.206
Depression				0.81	0.32–2.08	0.662
Diabetes				0.94	0.39–2.23	0.884
Hypertension				1.21	0.76–1.91	0.425
Smoke				2.04	1.47–2.82	< 0.001
Drink				1.18	0.79–1.76	0.420
**Low birth weight**						
Violent crimes						
1st quintile	Ref.					
2nd quintile	1.38	1.00–1.91	0.050	1.37	0.98–1.91	0.063
3rd quintile	1.32	0.88–1.99	0.178	1.36	0.89–2.07	0.153
4th quintile	1.48	1.10–1.99	0.010	1.50	1.11–2.05	0.009
5th quintile	1.42	1.04–1.95	0.029	1.42	1.03–1.98	0.034
Physical violence				2.12	1.03–4.36	0.042
Depression				0.71	0.22–2.25	0.557
Diabetes				0.30	0.07–1.30	0.107
Hypertension				1.73	1.10–2.71	0.017
Smoke				2.89	2.07–4.03	< 0.001
Drink				1.14	0.77–1.69	0.522

OR: odds ratio; 95% CI: 95% confidence interval; All analyses used multiple imputations. Imputed data were averaged across the 50 imputed data sets using Rubin’s rule [41]. *Adjusted for maternal age, child sex (female), education, and socioeconomic status

## Discussion

This study uses a novel data set from Brazil to explore the empirical associations between neighborhood-level exposure to violent crime and birth outcomes. Our study yields three main findings: first, exposure to violent crimes is very high among pregnant women in the urban areas studied. Based on the São Paulo estimated population by the Brazilian Institute of Geography and Statistics and violent crimes reports extracted by us from the Secretariat of Public Safety of the State of São Paulo, from 2011 to 2014, there were on average 2.87 violent crimes per 10,000 inhabitants in the city of São Paulo and an average of 3.62 violent crimes per 10,000 inhabitants in the study area. While these numbers may be somewhat abstract, they imply that each person living in a densely populated area like the one studied - with an estimated density of 7770 inhabitants per km^2^ - experiences, on average, 0.94 violent crimes per month within 1-km walking distance from home. This implies 5.6 crimes in close vicinity within the first six months of pregnancy for the average pregnant mother. Even mothers will, with all likelihood, personally witness only a small fraction of these events, the threat of external violence is, without any doubt, real and constantly present.

Second, our results suggest that external violence should not be interpreted as homogeneous characteristics of entire cities or larger census tracts, but something highly heterogeneous within relatively small spatial areas. As shown in Fig. 1, the distance between some of the safest and some of the most violent exposed neighborhoods is less than 2 km, highlighting the remarkable small scale at which urban disparities can be detected.

Third, and maybe most importantly, we found that exposure to crime is associated with substantial SGA, PT, and LBW increases even after controlling for SES status and maternal education, and other risk factors. Our findings that violence increases adverse birth outcomes are consistent with a previous municipality-level comparison from Brazil [27] and highlight the critical long-term consequences of violence beyond the immediate impact on the directly affected population. However, in their study, the authors found a pronounced difference among children of poorly educated mothers, suggesting that violence is only a component of the disadvantages that these children are exposed to due to low SES [27]. Similarly, another study found that neighborhood-level socio-economic-related risks directly affect LBW and PT, and these effects are mediated by personal-level risks such as SES status [17]. Despite the evidence, the pathways connecting neighborhood-level social and environmental conditions to individual-level adverse birth outcomes remain poorly understood. Differences in birth outcomes across different socioeconomic levels could be due to psychosocial stressors [17, 54], which could trigger adverse birth outcomes through a series of behavioral and neuroendocrinal interactions affecting the intrauterine environment [7, 15–19, 21–23, 55].

The general literature on violence exposure and birth outcomes has been more mixed with some studies such as Messer et al. [26], finding no associations with adverse birth outcomes and others such as Goin et al. [29], finding only an association between first-trimester exposure and SGA. While several theoretical mechanisms highlighted in the literature and introduction of this paper seem consistent with the results presented here, we do not have data to directly investigate mechanisms such as blood pressure increases [56] and anxiety [57] highlighted in the literature. HPA axis activation would be consistent with the PT delivery increase documented in several epidemiological studies [20–22, 58, 59]. It is also possible that heightened maternal cortisol levels may lead to increased placental corticotrophin and prostanoid release. This can stimulate uterine contractions, induce preterm delivery [17, 20–23], and cause intrauterine growth restriction [7]. Although placental enzymes protect the fetus from increased maternal cortisol levels in principle, animal studies suggest that prenatal stress can affect the placental activity and in utero enzyme expression [60, 61].

Our results corroborate the previous literature and add to the understanding of violence and its influence on health in a densely populated and heterogenous setting in an LMIC. Although our study did not explore the direct violence against pregnant women, previous findings suggest that domestic violence against pregnant women and mental disorders during pregnancy can have detrimental effects on birth outcomes in the Western Region of São Paulo [62]. The pathways between psychosocial stressors, like indirect and direct violence during pregnancy, and adverse birth outcomes, are poorly understood. Still, both negatively affect maternal and child health, possibly, by a common pathway.

Rapid rates of urbanization combined with increasing economic and gender inequalities are likely to trigger increased rates of violent crime and lead to a higher prevalence of risk factors in many settings, especially in areas where access to basic education and health care services remains limited [63, 64]. High violence rates may also endorse and strengthen social and cultural norms around masculinity and gendered power relationships [65]. Intersectoral strategies to decrease violence are urgently needed, and health programs must be improved to support victims of all kinds of violence.

### Limitations

The analysis presented in this paper has some limitations. The ROC-cohort enrolled mother-child dyads at the delivery, and important risk factors such as diabetes, hypertension, and physical violence were assessed only through the postpartum questionnaire. Those variables were in the analysis as “yes/no” did not assess the extent or in which period of the pregnancy it occurred. Moreover, smoking and drinking were assessed in five categories ranging from “never” to “every day”, and in both variables, almost 90% fell in the first category (never). Therefore, we decided to recode to “yes/no”, since we also could not know which period of the pregnancy occurred. Another limitation is that in our post-partum questionnaire we did not collect information related to intimate partner abuse (physical, emotional, financial), rape, and child sexual assault which could influence women’s sense of safety that could impact pregnancy outcomes.

Although violence exposure during the third trimester of pregnancy is likely to trigger adverse birth outcomes, our study only considered violence exposure during the first two trimesters of pregnancy. The primary reason for this was the presence of early preterm children (24–28 gestational weeks) in our sample, which did not allow us to compute third-trimester exposure variables. Excluding these cases or computing only partial exposures would likely have resulted in substantial estimation bias. It is also not clear how complete the violence data is. The National System of Statistics on Public Security and Criminal Justice [66] classifies each State as having low, medium, or high-quality data, according to the extent of its data coverage and the proportion of deaths with undetermined causes, and São Paulo is in the high-quality group data. It is possible that our data still underestimates the number of crimes in the area because of non-reported or non-registered crimes because, in disadvantaged areas, the residents tend to report only the most salient crimes. However, we believe that this type of measurement error should be small in the studied setting due to the extensive policing and the relatively high economic development levels.

## Conclusions

The results presented in this paper suggest that living in a neighborhood with high rates of violent crime is associated with substantial increases in small-for-gestational-age, preterm and low birth weight births. Actions to improve neighborhood safety and to prevent violence could reduce inequalities among disadvantaged populations with likely substantial long-term benefits on population health and wellbeing.

## Supplementary Information

**Additional file 1 Supplementary file 1:** Postpartum questionnaire.

**Additional file 2 Supplementary file 2:** Multiple imputations. **Table S1.** Missingness among variables used in the multiple imputation models.

## Data Availability

The violence information used in this study is publicly available at the Secretariat of Public Safety of the State of São Paulo webpage: www.ssp.sp.gov.br. The individual data that support the findings of this study include personal information such as residential address and health-related data. Therefore, the data are not publicly available. The data can be made available upon reasonable request to the corresponding author after obtaining permission from the University Hospital of the School of Medicine of the University of São Paulo’s Ethics board in accordance with the ethics policy statements related to the study protocol.

## Abbreviations

HIC: High-income countries
LMICs: Low-middle-income countries
ROC-Cohort: Western Region Birth Cohort (*Região Oeste Coorte*)
LBW: Low birth weight
PT: Preterm delivery
SGA: Small-for-gestational-age
SES: Socioeconomic status
PCA: Principal component analysis
MICE: Multiple imputations by chained eqs.
95%CI: 95% confidence interval
RRR: Relative Risk-Ratio
SD: Standard deviation
OR: Odds ratio
