# Supplementary material for: Associations between neighborhood violence during pregnancy and birth outcomes: evidence from São Paulo’s Western Region Birth Cohort
Source: BMC Public Health. 2021 May 5;21:865. doi: 10.1186/s12889-021-10900-y (PMC8097258; doi:10.1186/s12889-021-10900-y)
Supplement: Supplementary file 1 — Additional file 1 Supplementary file 1: Postpartum questionnaire. [file 12889_2021_10900_MOESM1_ESM.docx]

**Supplementary file 1: Postpartum questionnaire**

Angelica Carreira dos Santos¹^*^, Alexandra Brentani¹, Günther Fink²

**^*^Correspondence:**

angelica.carreira@gmail.com

^1^Angélica Carreira dos Santos, PhD

Department of Pediatrics, University of São Paulo Medical School, Av. Dr. Enéas Carvalho de Aguiar, 647, São Paulo, CEP- 05403-000, Brazil.

This questionnaire was developed to be used in this research and applied during the postpartum hospital stay by trained interviewers.

**Questionnaire:**

*[Instructions to the interviewer: Please request consent by means of IC before applying the questionnaire]*

| **Q1** | The mother agrees to participate | 1 Yes; 2 No: End interview |
| --- | --- | --- |
| **Q2** | What is your full name? | __________________ |
| **Q3** | What is your registration HU? | __________________ |
| **Q4** | What is your date of birth? | Day / Month / Year |
| **Q5** | Is your baby alive? | 1 Yes; 2 No: to skip Q5 |
| **Q6** | What is the baby’s name? | __________________ |
| **Q7** | It is boy or girl? | 1 Boy; 2 Girl |
| **Q8** | What is the height of the baby? | ____ cm |
| **Q9** | What is the weight of the baby? | ____ kg |
| **Q10** | What is your mother's name? | ___________________  First Name Last Name |
| **Q11** | What is your father's name? *[Put DK if unknown]* | ___________________  First Name Last Name |
| **Q12** | What is your height? | ____ cm |
| **Q13** | What was your weight before becoming pregnant? | ____ kg |
| **Q14** | What is your current weight? | ____ kg |
| **Now let me ask a few questions about pregnancy:** | | |
| **Q15** | What was the day of delivery? | Day / Month / Year |
| **Q16** | What was the expected date of delivery? | Day / Month / Year |
| **Q17** | How many previous births have you had *[put "0" if you've never given birth before;* *twins count as one pregnancy]* | Children ___  Pregnancies ____  Abortions ____ |
| **Q17a** | Do you have a previous history of loss of children? | Stillborn ____ |
| **Q18** | Was this pregnancy planned? | 1 Yes; 2 No |
| **Q19a** | Where did you do pre-natal care? | 1 Private Insurance  2 Did not. Skip to Q21  3 Basic Health Unit. Unit Name:  4 Family Health Care. Team color:  5 Other |
| **Q19** | Do you remember at how many weeks / months you began prenatal care? | _______ |
| **Q20** | How many prenatal visits did you make in the clinic?  [Interviewer*: just make sure to account for prenatal consultations, if the mother is not sure of the number, ask month]* | _______ |
| **Now let's talk about some issues that women may experience during pregnancy. Please say if you had each issue during this pregnancy:** | | |
| **Q21** | Diabetes | 1 Yes; 2 No |
| **Q22** | Hypertension | 1 Yes; 2 No |
| **Q23** | Depression | 1 Yes; 2 No |
| **Q24** | How often did you smoke during pregnancy? | 1 Never  2 Very rarely (<5 times)  3 At least once a month  4 At least once every week  5 Every day Average number of cigarettes |
| **Q25** | How often did you consume alcohol during pregnancy? | 1 Never  2 Very rarely (<5 times)  3 At least once a month  4 At least once every week  5 Everyday |
| **Q26** | How often have you used illicit drugs during pregnancy? | 1 Never  2 Very rarely (<5 times)  3 At least once a month  4 At least once every week  5 Everyday |
| **Q27** | Have you suffered from any physical violence during pregnancy? | 1 Yes; 2 No |
| **Q27a** | Have you or a family member been arrested/have a criminal record or were locked up at the police? | 1 Yes: Who? _____  2 No |
| **Q27b** | How would you rate your relationship during pregnancy? | 1 Very conflicted  2 Quarrelsome  3 Somewhat conflicting  4 A little conflicted  5 Not conflicted |
| ***Now I will ask questions about your parents and siblings*** | | |
| **Q28** | How long has your mother studied? | 1 Illiterate  2 Incomplete primary  3 Primary school (1st to 9th)  4 Middle school (high school)  5 Completed higher education |
| **Q29** | How long your father studied? | 1 Illiterate  2 Incomplete primary  3 Primary school (1st to 9th)  4 Middle school (high school)  5 Completed higher education |
| **Q30** | How many brothers and sisters do you have? Are all alive? | __#brothers #deceased: ___  __#sisters # deceased: ___ |
| **Q31** | What was the economic status of his family during his childhood? | Class A - Up to R$ 12,440.00  Class B - Over R$ 6,220.00 to R$ 12,440.00  Class C - Over R $2,488.00 to R$ 6,220.00  Class D - Over R$ 1,244 to R$ 2,488.00  Class E- Up to R$ 1,244.00 |
| **Now let's talk a bit about yourself:** | | |
| **Q32** | What is your education level? | 1 Illiterate  2 Incomplete primary  3 Primary school (1st to 9th)  4 Middle school (high school)  5 Completed higher education |
| **Q33** | Before pregnancy, what was your occupation? | ______________ |
| **Q34** | What is your marital status? | 1 Single  2 Married  3 Divorced  4 Other |
| **Q35** | Including you, how many adults (> 18 years) are living in your home today? | ____ |
| **Q35 to** | Number of children (<18 years) living in your home today? | ____ |
| **Q36** | How many rooms does your household have? | ___ Rooms |
| **Q37** | From what material it is constructed? | 1 Wood  2 Block  3 Apartment |
| **Q38** | What is the total monthly income of your family?  [If you are unsure, individual income] | Up to R$ 622.00 (minimal wage)  Up to R$ 1,244.00  Up to R$ 2,488.00  Up to R$ 6,220.00  up to R$ 12,440.00  over R$ 12,440.00 |
| **Q39** | Do you currently pay rent? | 1 Yes  2 No. I own a property.  3 No. I live with my parents.  4 Other: ___________________ |
| **Q 40** | If you pay rent, what is the monthly fee? | R$ ________ |
|  | Does your family receive the *Bolsa Familia* (governmental program)? | 1 Yes; 2 No |
| **Q41** | How many cars do your family have? | _____ |
| ***As part of this project, we use data from medical records of your baby, do you authorize?*** | | |
| **Q42** | Authorize | 1 Yes; 2 No |
| ***In the future, we intend to monitor the development of your baby in home visits, do you authorize?*** | | |
| **Q43** | Authorize | 1 Yes; 2 No: End interview |
| **You give us contact information so we can look for it later?** | | |
| **Q44** | Current Address: |  |
| **Q45** | Current phone number: |  |
|  | E-mail |  |
| **Q46** | Present address of parent |  |
| **Q47** | Current phone number of mother: |  |
| **Q48** | In addition to his mother, who can we contact if you change your address? | ______________________  First Name Last Name |
| **Q49** | Address: |  |
| **Q50** | Phone number: |  |
|  | E-mail: |  |
| **Q51** | Name of Second Contact |  |
| **Q52** | Address: |  |
| **Q 53** | Phone number: |  |
|  | E-mail: |  |
| **Thank you for your cooperation!** | | |
|  | | |

*[End of the questionnaire]*
