# Supplementary material for: Associations between neighborhood violence during pregnancy and birth outcomes: evidence from São Paulo’s Western Region Birth Cohort
Source: BMC Public Health. 2021 May 5;21:865. doi: 10.1186/s12889-021-10900-y (PMC8097258; doi:10.1186/s12889-021-10900-y)
Supplement: Supplementary file 2 — Additional file 2 Supplementary file 2: Multiple imputations. Table S1. Missingness among variables used in the multiple imputation models. [file 12889_2021_10900_MOESM2_ESM.docx]

**Supplementary file 2: Multiple imputations**

Angelica Carreira dos Santos¹^*^, Alexandra Brentani¹, Günther Fink²

**^*^Correspondence:**

angelica.carreira@gmail.com

^1^Angélica Carreira dos Santos, PhD

Department of Pediatrics, University of São Paulo Medical School, Av. Dr. Enéas Carvalho de Aguiar, 647, São Paulo, CEP- 05403-000, Brazil.

We implemented multiple imputations due to the incomplete responses in the birth records as well as the postpartum questionnaires. We performed multiple imputations by chained equations (MICE) using Stata’s *mi* package [51]. The variables used in the MICE models included exposures (e.g., violence during pregnancy), birth outcomes (preterm, low birth weight and small for gestational age), maternal characteristics (e.g., age, income, education), pregnancy variables (e.g., diabetes, hypertension), and neonatal variables (e.g., birth weight, length, and sex). We generated 50 independent datasets (M = 50) and followed Rubin’s rules to aggregate results across imputed datasets [52].

**Table S1.** Missingness among variables used in the multiple imputation models.

| Variable | % missing values | Type of data | Model for data prediction |
| --- | --- | --- | --- |
| Violent crimes | 0 | Categorical (5 categories) | No missing |
| LBW | 0 | Binary | No missing |
| PT | 0 | Binary | No missing |
| Sex (Female) | 0 | Binary | No missing |
| Birth weight | 0 | Continuous | No missing |
| Gestational age (weeks) | 0 | Continuous | No missing |
| Maternal age | 0 | Continuous | No missing |
| Skin color (white) | 0 | Binary | No missing |
| Birth length | 3.3 | Continuous | Linear regression |
| SGA | 3.6 | Binary | Logistic regression |
| Maternal education | 38.2 | Categorical (3 categories) | Multinomial logistic regression |
| Hypertension in pregnancy | 38.3 | Binary | Logistic regression |
| Depression in pregnancy | 38.3 | Binary | Logistic regression |
| Diabetes in pregnancy | 38.4 | Binary | Logistic regression |
| Drink in pregnancy | 38.4 | Binary | Logistic regression |
| Smoke in pregnancy | 38.5 | Binary | Logistic regression |
| Physical violence in pregnancy | 38.6 | Binary | Logistic regression |
| Socioeconomic status | 48.6 | Categorical (5 categories) | Multinomial logistic regression |

_LBW: low birth weight; PT: preterm; SGA: small for gestational age._
